# Head and neck cancer: a comprehensive review of epidemiology, global burden and advances in radiotherapy

**DOI:** 10.1186/s43046-026-00398-z

**Published:** 2026-08-22

**Authors:** Karunya A S, Sudeep Kumara K, Avin Kumar, Kamalaksh Shenoy

**Affiliations:** 1 Department of Physics, School of Engineering, Dayananda Sagar University, Bengaluru, 562112 India; 2 Department of Radiation Oncology, A J Hospital and Research Centre, Mangalore, India

**Keywords:** HNC, Epidemiology, Global burden, ASIR, ASMR, Risk factors, Radiotherapy

## Abstract

Head and neck cancer (HNC), a heterogeneous group of malignancies that arising from the mucosal surfaces of the Upper Aerodigestive Tract (UADT), is a major health concern worldwide. By 2050, the number of cancer cases worldwide is expected to reach 35 million, with nearly half of all new cases and deaths occurring in Asia. In India, HNC is particularly prevalent accounting for about 30% of the global burden, with lip and oral cavity cancers (OCC) make up 10.17% of all cases, highlighting a serious public health crisis. A comparative analysis of GLOBOCAN 2022 data reveals a significant variation in HNC burden; India’s overall Age-Standardized Incidence Rate (ASIR) for lip and OCC is 9.8, which is considerably higher than the Asian average of 3.2 and the global average of 4.1. Indian males facing a particularly high burden (ASIR: 14.7) compared to females (ASIR: 4.9). While India leads in sites such as the larynx and hypopharynx, it maintains a notably lower burden of nasopharyngeal cancer (ASIR: 0.4) compared to the rest of Asia (ASIR: 1.9). High Mortality-to-Incidence Ratio (MIR) persist across all sub-sites, with tobacco, alcohol, and Human Papillomavirus (HPV) being the main risk factors. This review critically analyses HNC literature by integrating historical perspectives with recent progress in radiation therapy (RT). This includes Three- Dimensional Conformal Radiation Therapy (3DCRT), Intensity- Modulated Radiation Therapy (IMRT), Image Guided Radiation Therapy (IGRT), Volumetric Modulated Arc Therapy (VMAT), and new methods like Stereotactic Radiosurgery (SRS), Stereotactic Radiotherapy (SRT), Stereotactic Body Radiation Therapy (SBRT), Tomotherapy, Proton therapy (PT) and Adaptive Radiotherapy (ART). Radiotherapy remains a cornerstone of treatment, with its success relying on precise technology and accurate treatment delivery. By analysing epidemiology, risk factors, and evolving clinical approaches, this work creates a clear framework for current management and highlights future directions to improve patient quality of life and treatment results.

## Introduction

Cancer is one of the top causes of death around the world. It causes a serious impact on society, health, and the economy. Asia plays a major role in the global cancer issue, with an estimated 9.8 million new cases and 5.4 million deaths each year, representing nearly half of all cases and deaths worldwide [1].

Head and neck cancer (HNC) includes a diverse group of tumors, arising from the mucosal surfaces of the Upper Aerodigestive Tract (UADT), such as the oral cavity, pharynx, and larynx [2, 3]. Together, these cancers make up a significant part of the global cancer burden and are a leading cause of morbidity and mortality around the world [4]. The incidence and distribution of HNC vary widely across regions, which reflects differences in exposure to known risk factors such as tobacco use (both smoking and chewing), alcohol consumption, Betel Quid (BQ) chewing, viral infections including HPV, and other environmental factors [4, 5]. In India, HNC is especially common, with lip and Oral Cavity Cancers (OCC) accounting for 10.17% of all cases according to GLOBOCAN, 2022, making them the most prevalent cancer type in the region and highlighting the disease as a serious public health concern [6].

In the past, the management of HNC has continued to improve with advancements in surgical, systemic, and radiation therapy modalities. Early treatment approaches were often associated with limited disease control and significant treatment-related toxicities [7, 8]. However, with advancements in diagnostic imaging and staging systems, management of HNC has continued to improve. Despite these advancements, achieving optimal tumor control while preserving organ function and quality of life remains a central challenge in HNC treatment [9].

Radiation therapy (RT) plays a pivotal role in the HNC, either as an adjuvant or as the primary mode of treatment, in combination with chemotherapy and surgery [7]. The complex anatomy of the head and neck region, along with the close proximity of critical organs at risk (OAR), demands high levels of precision in radiation delivery [10]. Technological advancements in RT have driven a transition from conventional two-dimensional radiotherapy (2DRT) to Three- Dimensional Conformal Radiation Therapy (3DCRT), and subsequently the advent of Intensity- Modulated Radiation Therapy (IMRT), and Image Guided Radiation Therapy (IGRT) [11, 12]. Recently, advanced techniques such as Stereotactic Radiosurgery (SRS), Stereotactic Radiotherapy (SRT), Volumetric Modulated Arc Therapy (VMAT), and Stereotactic Body Radiation Therapy (SBRT) in RT treatment plans, Tomotherapy, Proton Therapy (PT) and Adaptive Radiotherapy (ART), have further enhanced dose conformity and reduced exposure to surrounding normal tissues [13].

Alongside these technological advances, patient positioning and immobilization have received more attention as crucial elements of accurate radiotherapy delivery [14]. Reproducible positioning and image guidance are crucial because setup uncertainties and patient motion can compromise target coverage and increase the risk of toxicity [15]. To address these challenges, innovations like multi-axis treatment couches and sophisticated immobilization devices have been introduced.

The purpose of this review is to present a historical background and comprehensive review of the literature on the subject of HNC, focusing on the epidemiology, global disease burden, treatment approaches, and latest advancements in the HNC treatment by the radiotherapy technique. By integrating historical background and the latest literature, this review seeks to highlight progress achieved so far in the management of patients with HNC.

## Epidemiology

### Global

Geographical variation, environmental exposures and socioeconomic factors all have an impact on the incidence and mortality of cancer, which is a significant global public health concern [16]. According to International Agency for Research on Cancer (IARC) estimates for 2022, cancer accounted for approximately 9.7 million deaths and 19.98 million new cases worldwide, including non-melanoma skin cancers. Projections suggest that global cancer incidence may reach 35 million cases annually by 2050, reflecting demographic growth and population aging [4].

**Fig. 1:**
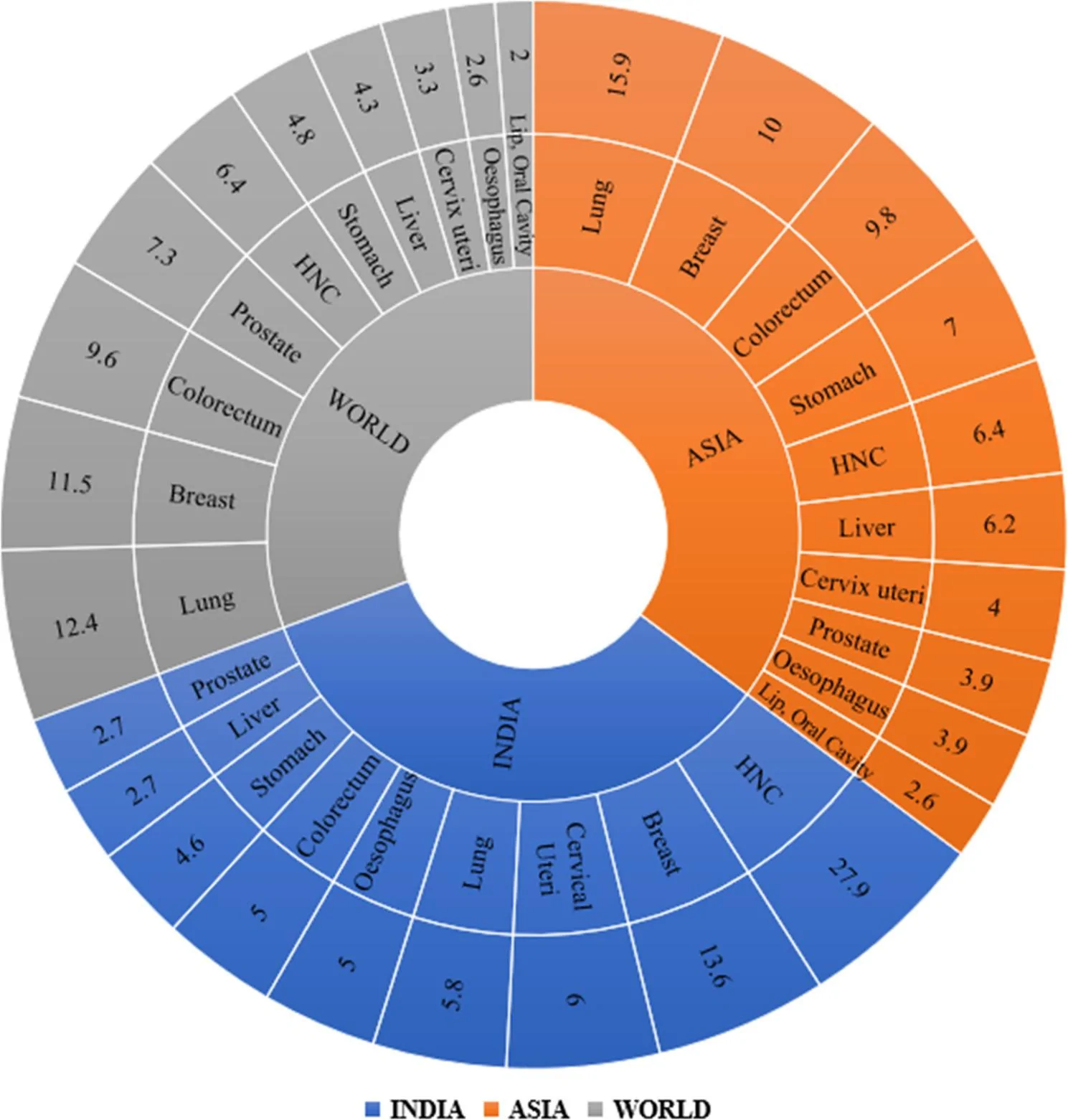
Percentage distribution of cancer incidence in India, Asia, and the World as reported in GLOBOCAN, 2022

Among major cancer sites, lung and breast cancers remain the most frequently diagnosed globally, while HNC constitutes a significant proportion of cases, particularly in low and middle-income countries. Based on the data available from the WHO (2022) occurrence of cancer in major sites is shown in Fig. 1.

### Asia

Asia is the most densely and diversely populated region, representing nearly 60% of the global population. Improvements in the healthcare system and continued socioeconomic growth have contributed to a significant increase in life expectancy in Asia. In 2022, an estimated 9.8 million incident cancer cases and 5.4 million deaths occurred across Asia, constituting nearly 50% of the global burden, thereby signalling a major public health threat in the region. China, India, and Japan collectively account for the highest cancer incidence and mortality across the Asian continent [1]. In Asia (2022), lung cancer ranks first, followed by breast cancer, colorectal cancer, stomach cancer, and HNC as the most commonly occurring malignancies.

**Fig. 2:**
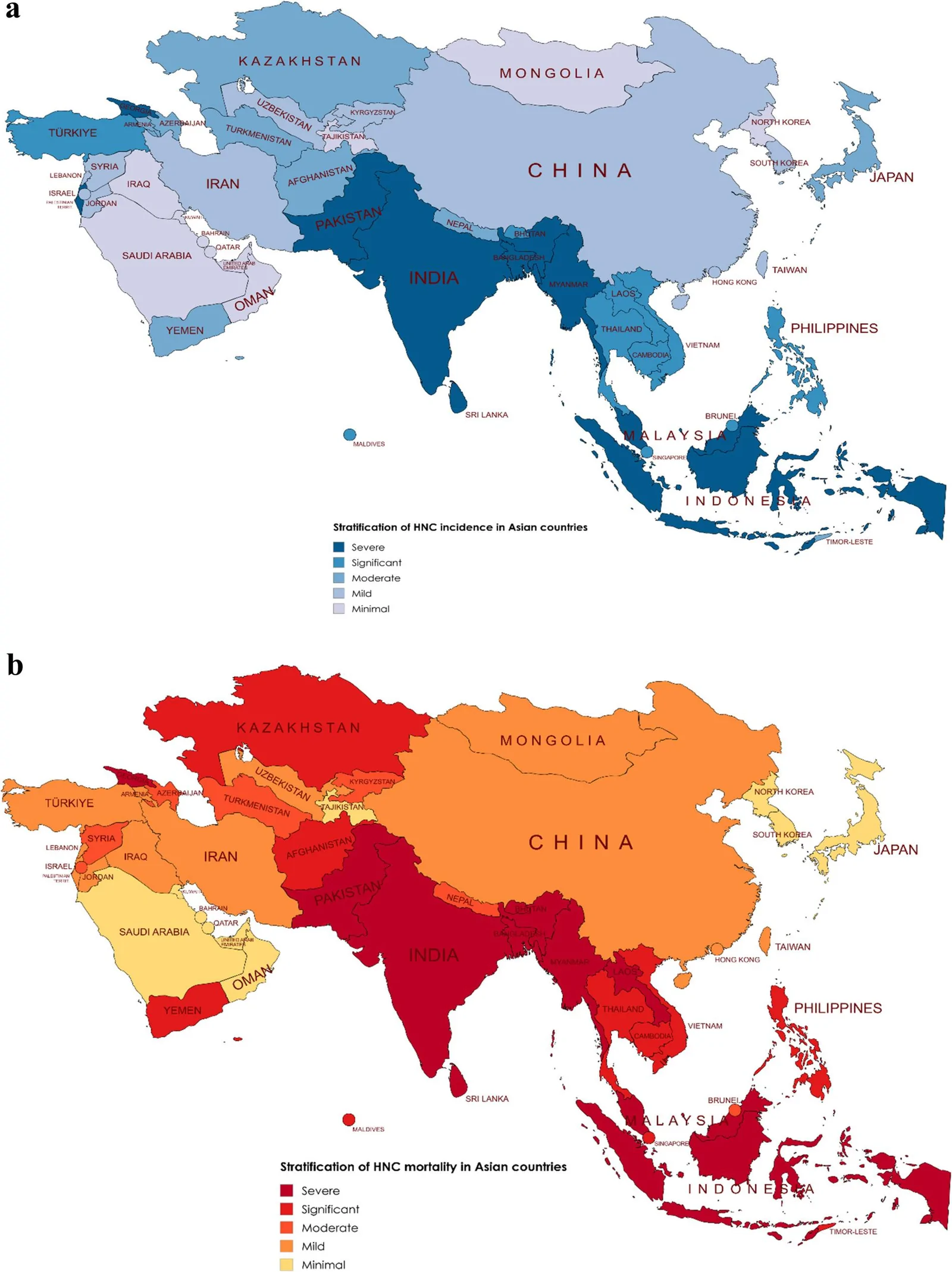
**a** Map showing estimated HNC incidence in Asia in 2022 (GLOBOCAN 2022). **b** Map showing estimated HNC mortality in Asia, in 2022 (GLOBOCAN 2022). (https://www.mapchart.net/asia.html)

As per the WHO (2022) data of Asia, it is noticed that the incidence rate (Fig. 2a) and mortality rate (Fig. 2b) of HNCs are significantly higher in India compared to other Asian countries. The rise in HNCs in Asia is mainly due to the widespread use of tobacco smoking and chewing accounts for up to 80% of all head and neck squamous cell carcinoma (HNSCC) cases [17]. Cultural habits like BQ, pan, gutkha, supari consumption, lack of oral hygiene, unawareness of early symptoms, poor screening, late-stage diagnosis, and limited access to specialized cancer care, especially in rural areas, including India’s largest population, make the countries a hotspot for HNC burden. Socio-economic factors, such as poverty, poor education, and high treatment costs, also contribute to delays in diagnosis and treatment. Additionally, exposure to environmental and occupational pollutants further increases risk, making HNC a major public health concern in Asia.

### India

**Fig. 3:**
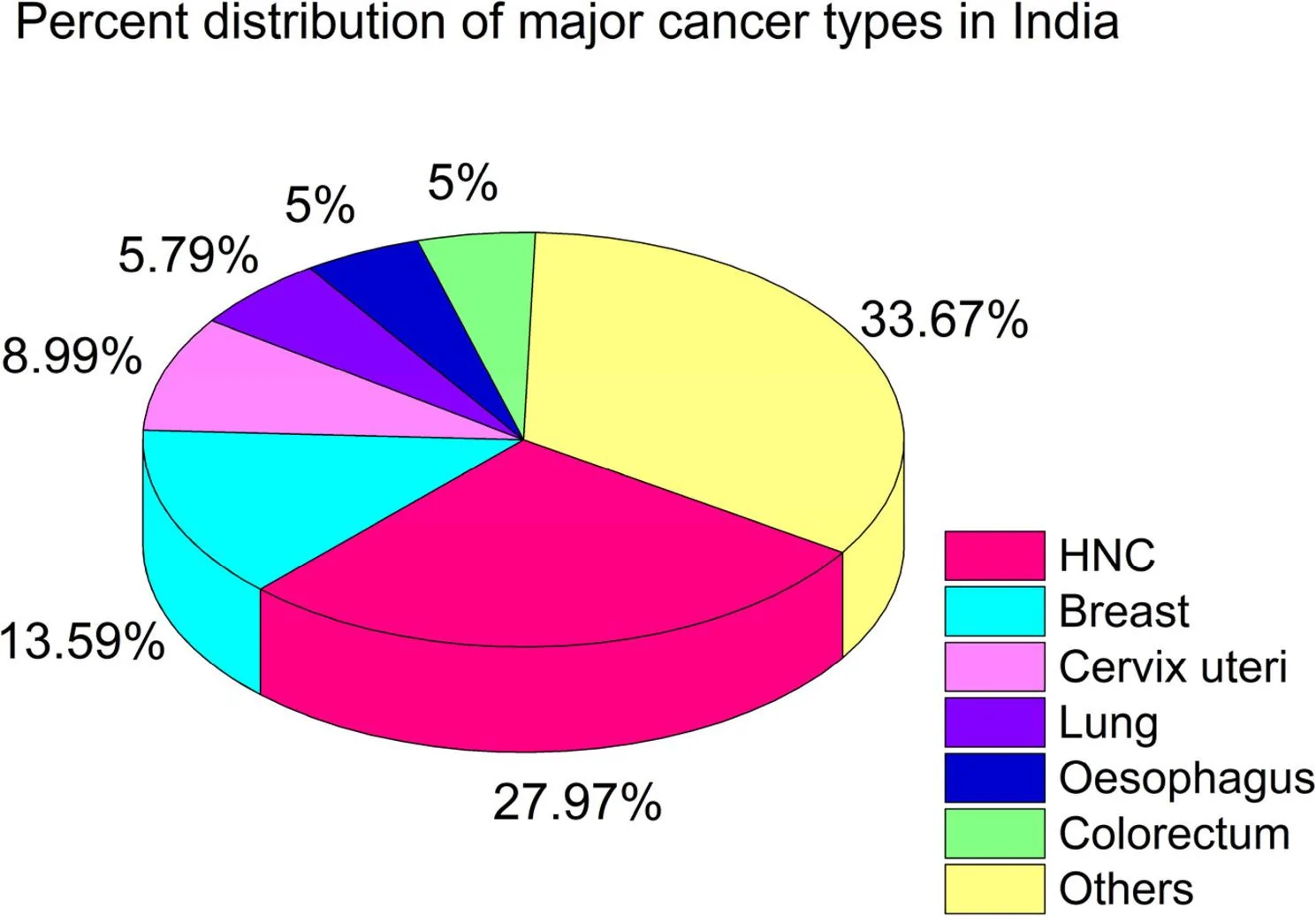
Percentage distribution of cancer incidence in India as reported in GLOBOCAN, 2022

Cancer occurrence is not homogeneous in India, but it is an increasing health challenge in the country, impacting lives across diverse socio-economic and geographies of the country [18]. In a population of 1.46 billion, nearly 1.3 million individuals develop cancer every year. Roughly one in every 10 Indians will develop cancer in their lifetime and this proportion may rise with improved detection and diagnosis. Mathur et al. [19] report that there were an estimated 13.9 million cancer cases in the country in 2020. India ranked third after China and the USA [20]. Among the top five cancers in the country, HNC (27.9%) was found to be the highest, followed by breast (13.6%), cervix uteri (9%), and lung (5.8%), this is diagrammatically shown in the Fig. 3. The cancer rise in India can be attributed to several determinants, including rapid urbanization, aging demographics, sedentary behaviour, and the widespread adoption of nutritionally poor diets. Moreover, the escalating exposure to both indoor and outdoor air pollution are becoming a major area of concern [21, 22]. The escalating rate of cancer diagnosis in India presents a formidable challenge to public health.

## Results and discussion

### Head and neck cancer

Head and Neck Cancer represents one of the most frequently diagnosed malignancies. These malignancies originate in the UADT, affecting the mucosal lining of the oral cavity, nasopharynx, oropharynx, hypopharynx, and larynx [2, 3]. Other less common sites include the skin of the head and neck area, the salivary glands, the nasal cavities, and the paranasal sinuses [2]. From a histopathological perspective, more than 90% of all cancers in the head and neck region are squamous cell tumors. Men experience a higher incidence of HNC compared to women, and it typically arises between the ages of 50 and 60 years [2]. Cancer of different types of salivary glands can also begin in HNCs, but this category of HNC is comparatively infrequent. Depending on the site in which cancer begins, HNCs are classified into different cancer groups, namely, oral cavity, salivary glands, pharynx, larynx, nasal cavity, and paranasal sinus [23].

Head and neck cancers consists of a heterogeneous group of malignancies arising from anatomically distinct sites, each characterised by specific ICD-O-3 topography codes and varying epidemiological patterns. As presented in Table 1, the major HNC sites include the lip and oral cavity (C00–C06), larynx (C32), hypopharynx (C12–C13), oropharynx (C01, C05.1–C05.2, C09–C10), nasopharynx (C11), and salivary glands (C07–C08). The lip and oral cavity encompass the lips and commissures, tongue (excluding the base of the tongue), gingiva, floor of the mouth, hard palate, buccal mucosa, and retromolar region whereas the larynx comprises the supraglottis, glottis, and subglottis. The hypopharynx includes the pyriform sinus, postcricoid region and posterior pharyngeal wall, while the oropharynx comprises the tonsillar fossae and pillars, vallecula, lateral and posterior oropharyngeal walls, base of the tongue, and soft palate. The major salivary glands include the parotid, submandibular, and sublingual glands, whereas the nasopharynx comprises its anterior, superior, posterior, and lateral walls. Although these anatomical subsites are clinically distinct, GLOBOCAN 2022 does not provide subsite-specific ASIR or ASMRs; therefore, epidemiological estimates for the individual subsites could not be included.

**Table 1:** Anatomical subsites, ICD-O-3 codes, and ASIR and ASMR of HNCs in India, Asia, and world based on GLOBOCAN 2022

Types of HNC	Anatomical subsite of HNC	ICD-O-3 Codes	ASIR (100,000)			ASMR (100,000)		
			India	Asia	World	India	Asia	World
Lip & Oral cavity	• Lips & Commissures, • All surfaces of the tongue except the base of tongue, • Gingiva (upper and lower), • Floor of mouth, • Hard palate, • Buccal mucosa, • Retromolar trigone	C00 – C06	9.8	3.2	4.1	5.4	1.6	3.9
Larynx	• Supraglottis, • Glottis, • subglottis	C32	2.4	1.2	0.9	1.7	0.7	2.1
Hypopharynx	• Pyriform sinus, • Postcricoid region, • posterior pharyngeal wall	C12 – C13	2.1	0.6	1.2	1.6	0.4	0.8
Oropharynx	• Tonsillar fossae, • Tonsillar pillars (faucial arch), • Vallecula, • Lateral and posterior walls of oropharynx, • Base of tongue, • Soft palate	C01, C05.1 – C05.2, C09 – C10	1.5	0.7	1.3	1.1	0.5	1.1
Nasopharynx	• Anterior, superior, posterior, and lateral walls of nasopharynx	C11	0.4	1.9	0.6	0.3	1.1	1.6
Salivary glands	• Parotid, • Submandibular or submaxillary, • Sublingual glands	C07 – C08	0.6	0.5	2.0	0.3	0.3	0.5

ICD-O-3 Codes: C00 - C06: C00.0, C00.1, C00.3, C00.4, C00.6, C02.0, C02.1, C02.2, C02.3, C02.4, C03.0, C03.1, C04.9, C05.0, C06.0, C06.2. C32: C32.0, C32.1, C32.2. C12 – C13: C12.9, C13.0, C13.2. C01, C05.1 - C05.2, C09 - C10: C01.9, C05.1, C09.0, C09.1, C10.0, C10.2, C10.3. C11: C11.3, C11.0, C11.1, C11.2. C07 – C08: C07.9, C08.0, C08.1

Considerable geographical variation exists in the ASIR and ASMR of HNC subsite across India, Asia, and the world (Table 1). Lip and OCC exhibit the highest ASIR in India (9.8 per 100,000) which is considerably higher than the Asian (3.2 per 100,000) and global (4.1 per 100,000) averages, accompanied by a relatively high ASMR of 5.4 per 100,000 in India. Similarly, laryngeal, hypopharyngeal, and oropharyngeal cancers demonstrate higher incidence and mortality rates in India than in both Asia and the global population. In contrast, nasopharyngeal cancer has lower ASIR and ASMR in India, exhibits substantially greater disease burden in Asia. Salivary gland cancers contribute minimally to the overall HNC burden, accounting lowest incidence and mortality rates across all geographical region. These regional variations highlight the importance of subsite-specific epidemiological patterns, which are influenced by different environmental exposures, lifestyle factors, oncogenic viral infections, and healthcare access. The epidemiological burden of HNCs in India is discussed in the following section.

### Incidence of HNC in India

**Fig. 4:**
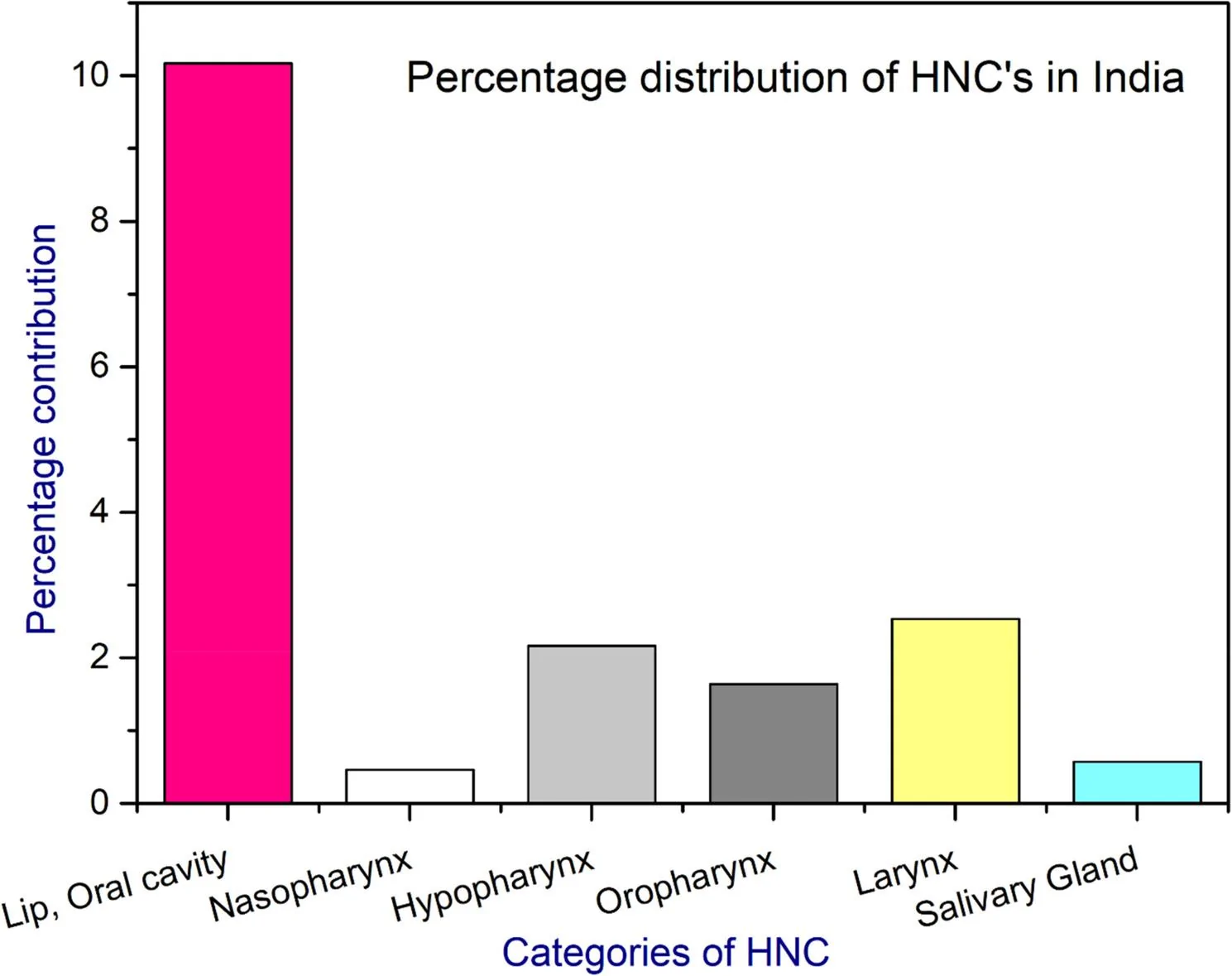
Percentage distribution of HNC incidence in India as reported in GLOBOCAN, 2022

Recent WHO statistics (2022) revealed that, HNSCC is the fifth most predominant cancer type globally, with the highest incidence and mortality rate being observed in the Asia region. Compared to other countries in Asia, India has seen a significant increase in the incidence of HNCs (Fig. 2a), accounting for approximately 30–40% of all cancer cases [24]. According to the IARC report, HNC statistics of India indicate an annual incidence of 2,51,613 cases, comprising 1,43,759 cases of lip and OCC, 35,855 of the larynges, 30,510 of the hypopharynxes, 23,174 of the oropharynges, 8,107 of the salivary glands, and 6,519 of the nasopharynges, respectively. The percentage distribution of HNCs in India is diagrammatically represented in Fig. 4. In men, around 28% of all cancers are HNCs, compared to 8% in women, with a lifetime risk of 1 in 33 for men and 1 in 107 for women. India experiences a higher HNC burden compared to the USA, UK, Australia, Africa, and Brazil [25]. More than 12,00,000 individuals were diagnosed with HNSCC around the world in 2022 [26]. However, in India, they generally include one-third of all the cancer cases and the number is anticipated to twofold by 2030. HNC is the commonest malignancy experienced in Indian males. Moreover, OCC is the foremost prevalent type among males and one of the most noteworthy over the globe. Across central, eastern, northern, southern, and western regions of India, mouth cancer has emerged as the predominant cancer site compared to the cancers of the tongue, larynx, and hypopharynx [25].

A number of factors play a role in elevating the risk of HNC. As per the Indian Council of Medical Research (ICMR), the sharp upward trend in HNC cases is due to extensive smoking and the use of tobacco, alcohol consumption, pan masala (which includes BQ, areca nut, and slaked lime), and gutkha [3, 24, 27]. Moreover, poor oral hygiene, micronutrient deficiencies, and HPV infection are reported to play a role in elevating the risk of HNC. In India, cases of oral and tongue cancers are elevated due to the widespread habit of tobacco chewing [28]. As previously mentioned, India’s global contribution of HNC patients is 57% [28]. Over the years, the rising use of tobacco has resulted in India accounting for nearly 60% of global HNC cases.

### Age-standardised incidence and mortality rates of HNC in India

**Fig. 5:**
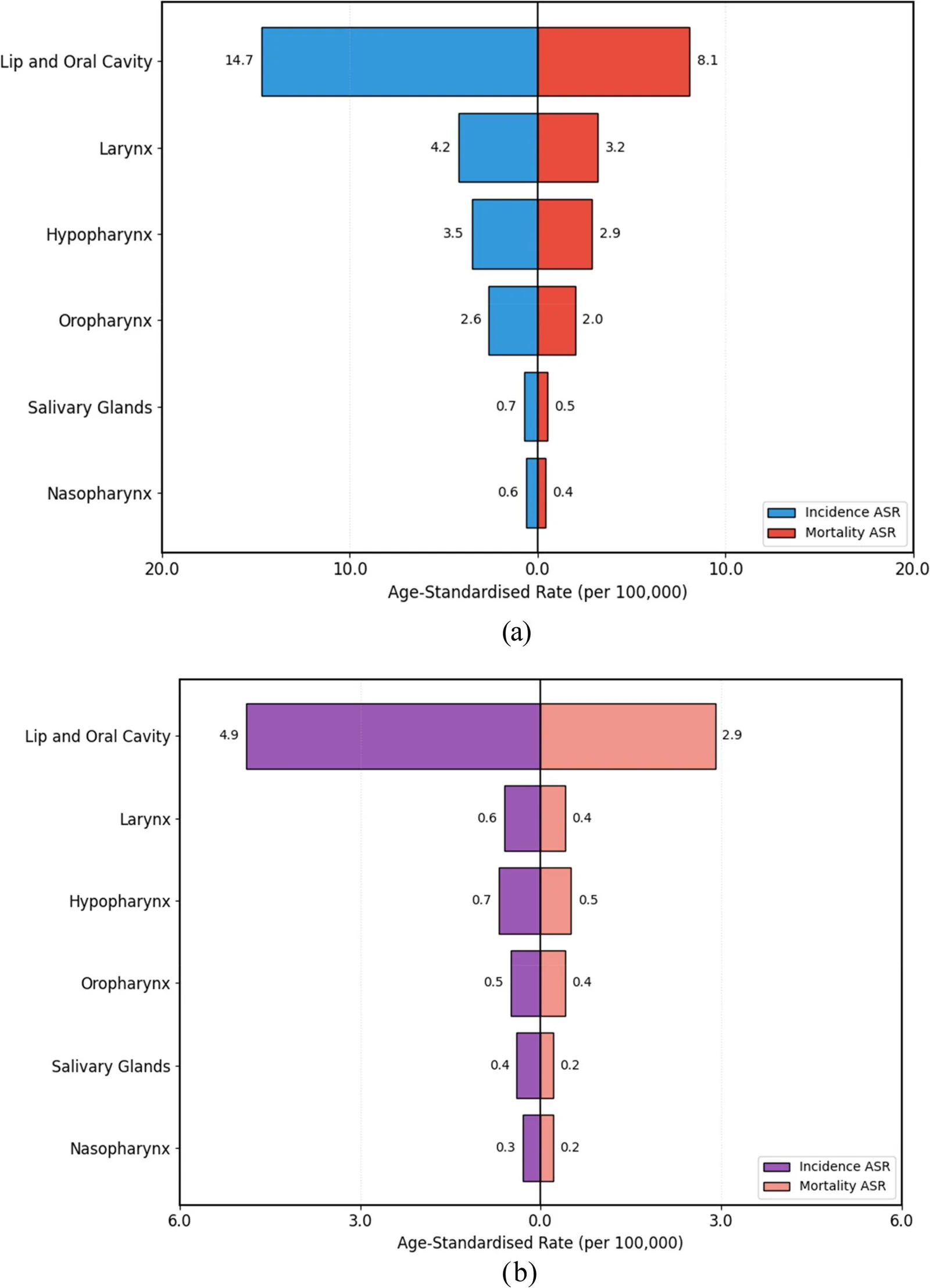
Estimated ASIR and ASMR in India for (**a**) Males and (**b**) Females

Figure 5 (a and b) illustrates the substantial burden of HNCs in India, revealing a significant gender disparity in disease distribution based on GLOBOCAN 2022 data [29]. Although both genders share similar high-risk sub-sites, the absolute burden is considerably higher in males [30]. Lip and OCC emerge as predominant sites for both genders; however, the ASIR for males (14.7) and ASMR (8.1) are approximately three times higher than female equivalents (ASIR: 4.9; ASMR: 2.9) in India [29]. Other important sites in males, like the larynx (4.2) and hypopharynx (3.5), exhibit significantly higher incidence rates than in females (0.6 and 0.7, respectively). This is probably due to males’ increased exposure to primary carcinogens such as tobacco use [29, 30].

A high Mortality-to-Incidence Ratio (MIR) is still present in both sexes especially in cases with late-stage oral diagnoses and pharyngeal cancers, indicating systemic difficulties in early detection and timely intervention [29]. With ASIR values below 1.0, minor salivary gland and nasopharyngeal cancers are still comparatively uncommon in both populations. This epidemiological profile highlights the urgent need for inclusive public health initiatives in India that guarantee all-encompassing care and encourage early screening and prompt detection based on the population’s distinct risk profiles [29].

### Comparison of ASIR and ASMR for HNC’s in India and Asia

**Fig. 6:**
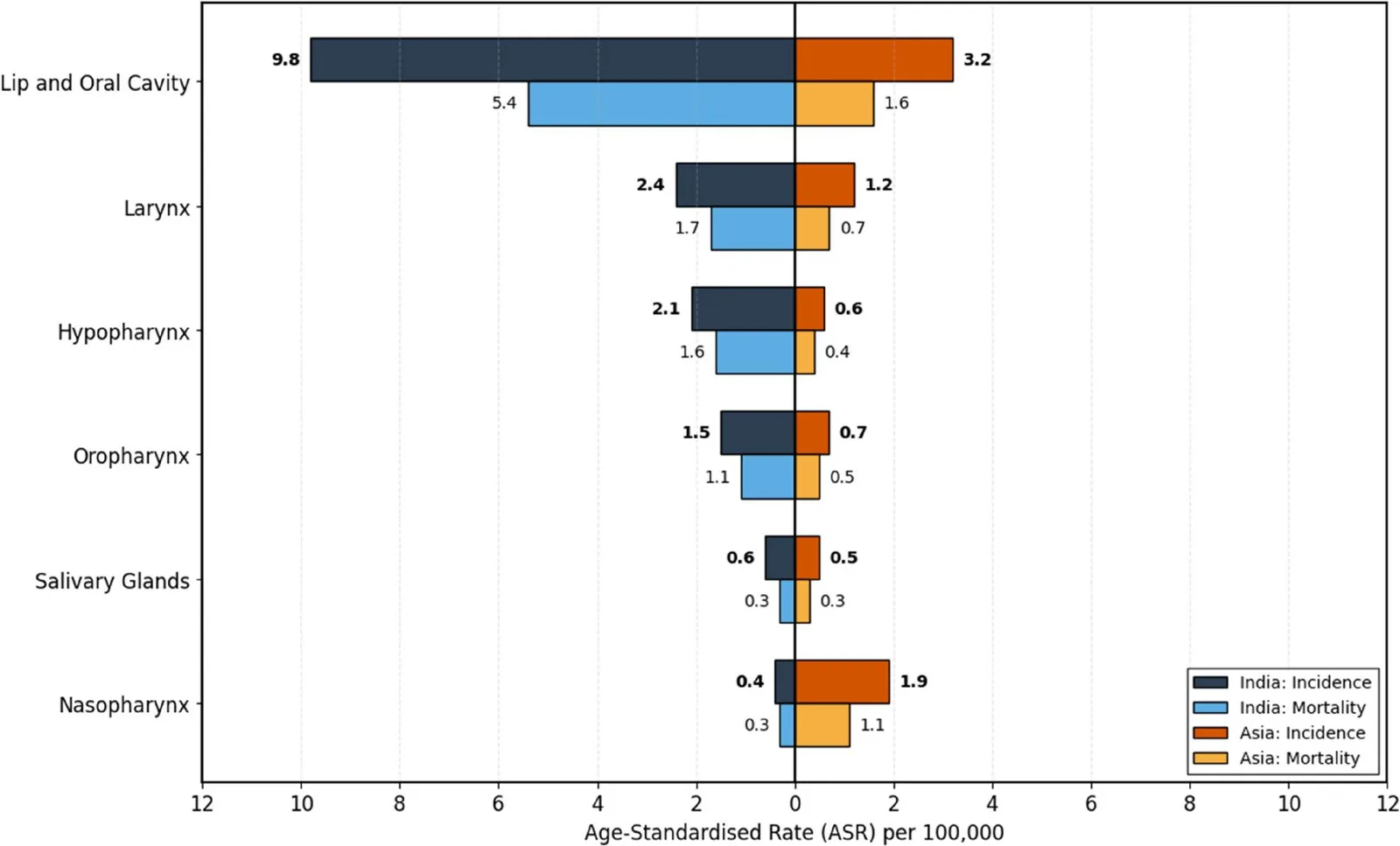
Estimated ASIR and ASMR for India and Asia

Based on GLOBOCAN 2022 data, the comparative analysis reveals a notable difference in the burden of HNC between India and Asia (Fig. 6). With an overall ASIR of 9.8, which is higher than the Asian average of 3.2, Lip and OCC are the most predominant subsite in India. Indian males bear a significantly higher burden with an ASIR of 14.7, nearly three times the rate seen in females (4.9), highlighting a critical public health crisis [29, 30].

India reports a notably lower burden of nasopharyngeal cancer with an overall ASIR of 0.4 compared to 1.9 for the rest of Asia, despite leading in the majority of HNC subsites, including the larynx and hypopharynx [31]. Across all subsites, MIR persist in India and Asia, indicating many cases are diagnosed at advanced, often fatal stages, emphasizing the need for aggressive, tailored tobacco cessation and early screening programs [32].

### The comprehensive risk profile of HNC in India

**Fig. 7:**
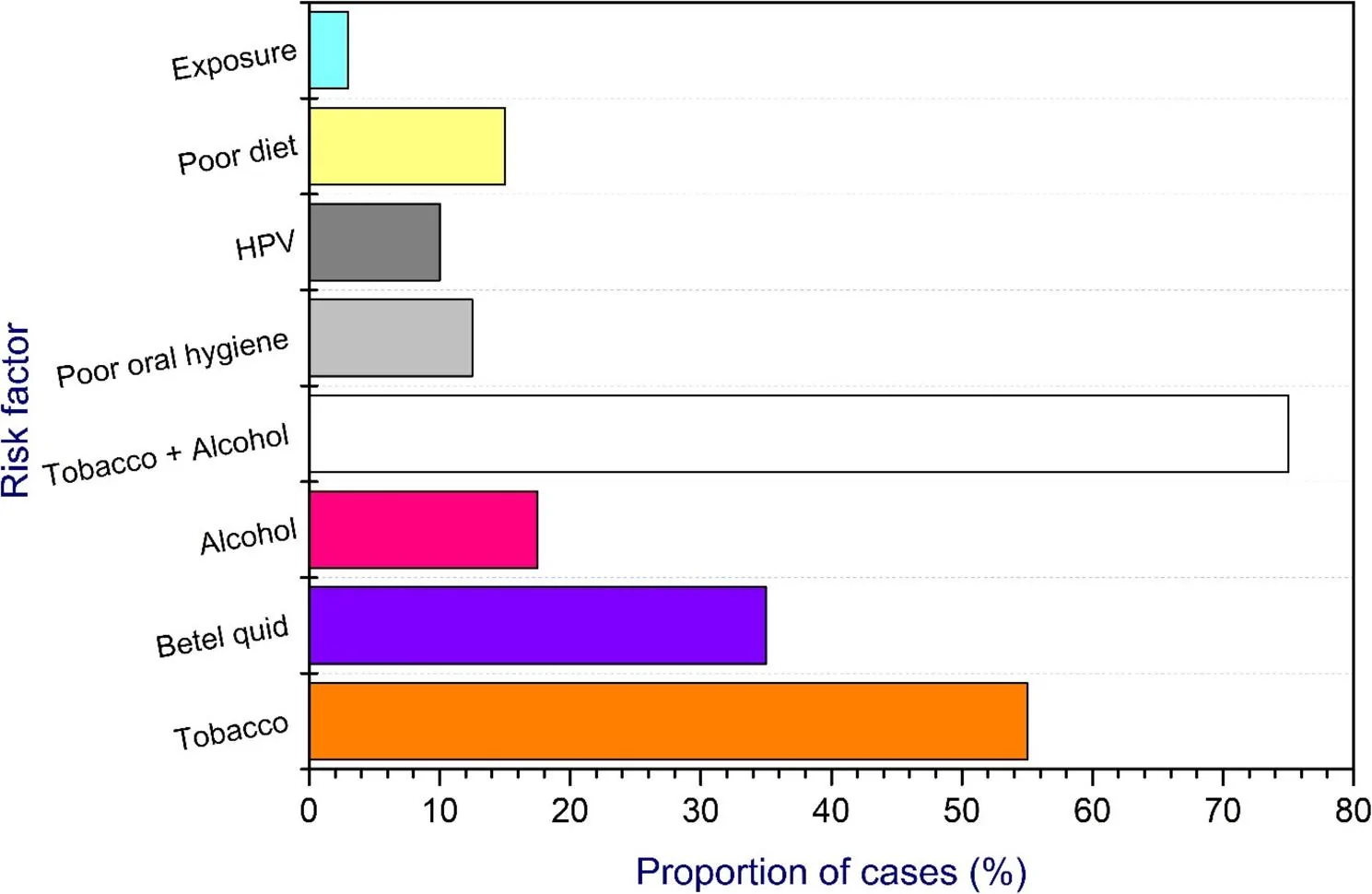
Risk factors of HNC in India

The prevalence of cancer is still rising dramatically worldwide. Tobacco use continues to be the primary cause of HNC, accounting for about 45% of cases globally. In India, however, tobacco and alcohol drive nearly 75% of HNC cases (Fig. 7); current data indicates that 38% of men and 9% of women aged 15 to 49 utilize high-risk products, such as bidis and smokeless tobacco [33]. Individuals who have used tobacco in chewing form are estimated to have an 80% higher risk of developing OCC [34].

Alcohol consumption is another critical independent risk factor, contributing to roughly 17.5% of HNC cases, particularly in the hypopharynx, with higher risks associated with potent spirits and heavy daily intake [35]. Individuals who both smoke heavily and drink frequently have a considerably higher risk of HNC, that are projected to be major contributors to the growing global incidence of HNSCC with the greatest impact seen in developing nations [36].

In addition to smoking and alcohol, the widespread habit of chewing BQ, is responsible for nearly 35% of HNC cases in India. BQ with tobacco, a traditional mixture of areca nut and lime, is a primary driver of the high HNC rates in India due to its potent carcinogenic properties [35, 37].

Infections like HPV now account for roughly 10% of cases; as of 2020, India contributed to 24% of all HPV related cancer cases globally. High-risk strains like 16 and 18, is a major driver of oropharyngeal cancers, specifically target the tonsils and the base of the tongue, often affecting younger populations regardless of tobacco history [38, 39].

**Fig. 8:**
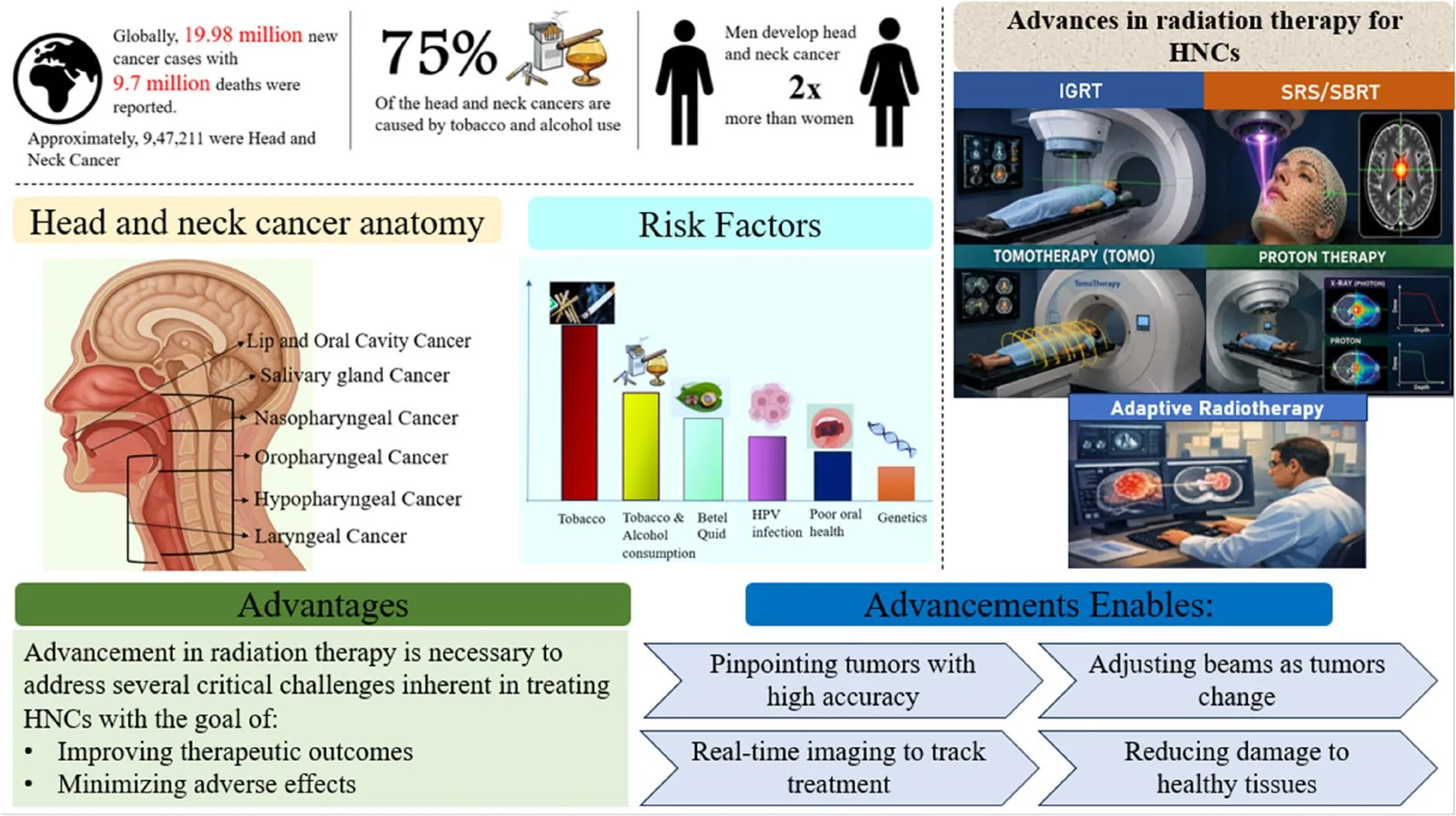
Overview of global burden, risk factors, anatomical sites, and advances in the management of HNC

Additionally, 12.5% of cases are caused by poor oral hygiene, which includes gum infections, missing teeth, and ill-fitting dentures. These conditions cause chronic physical trauma to the oral mucosa, which can trigger malignant transformations The risk spectrum completed by dietary habits and environmental exposures, with unhealthy diets linked to approximately 15% of cases [33]. An additional 3% of the burden comes from occupational hazards in sectors like construction and textiles where workers are exposed to formaldehyde, asbestos, and wood dust. When coupled with environmental factors like prolonged UV light exposure on the lips and genetic predispositions associated with Asian ancestry, these factors create a multifaceted public health challenge that necessitates targeted prevention strategies for the Indian population [33, 37]. The global burden, risk factors, anatomical sites, and advances in radiotherapy techniques for managing HNC are presented in the schematic Fig. 8.

### Advances in radiotherapy for HNC

The choice of the treatment is mainly based on the tumor stage, anatomical location, extent of disease and patients’ overall health and personal condition. Aim of the curative treatment is to completely eliminate cancer in order to achieve long-term survival or complete cure. It is commonly indicated for patients with early stage or advanced-stage HNCs. Successful curative treatment requires high dose of radiation delivered to the tumor with minimum irradiation of adjacent healthy tissues. Following are the various techniques used in curative treatment.

#### Conventional radiotherapy and three-dimensional conformal radiotherapy (3D-CRT)

Before 2000, RT was typically delivered using a simple conventional technique called 2DRT, which involved a single photon beam administered from two to four beam angles to treat the target volume [40]. However, this approach often resulted in significant side effects, particularly in HNC sites. Over the past three decades, radiotherapy planning has advanced from 2D to 3D image guided treatment planning. Even though 2DRT provided acceptable clinical outcomes, due to its limitations in target localization and normal tissue sparing resulted in the widespread adoption of 3D imaging based radiotherapy techniques for the curative management of HNSCC [26]. In 3DCRT, radiation beams are conformed to the shape of the tumor volume and an appropriate adjacent tissue margin is given to address microscopic disease spread. The design and delivery of RT treatment plans based on 3D image data with highly focused beams of radiation, allows precise dose to irregular tumour volume and reduces exposure to surrounding tissues when compared to traditional 2DRT [41].

#### Intensity modulated radiation therapy (IMRT)

Intensity modulated radiation therapy is the preferred radiotherapy technique for the HNC treatment, because of its complex anatomy and high risk of radiation induced toxicities. It is an advanced form of 3DCRT, that provides improved dose conformity around the irregularly shaped tumors while reducing the dose to the adjacent normal structures, introduced in the early 2000s [42]. It has become the standard method due to its ability to precisely target the primary tumor and regional lymph node while minimising the dose to brain, brain stem, optic nerves, parotid and salivary gland, thus achieving adequate target volume coverage and locoregional control [43].

#### Image guided radiation therapy (IGRT)

Image guided radiation therapy, another sophisticated advancement in the field of oncology, significantly utilizes imaging methods such as X-ray, CT, MRI with RT equipment to improve the precision in dose delivery. The core principle of IGRT is the integration of real-time imaging throughout treatment, allowing immediate corrections to beam alignment and patient positioning. This reduces margins around the tumor, minimising damage to healthy tissue. IGRT is now an integral part of RT delivery for HNC patients [44].

#### Volumetric modulated arc therapy (VMAT)

Although IMRT is widely used in the management of HNC treatment, concerns remain regarding the increased monitor units and prolonged treatment delivery times. VMAT is a technique that involves continuous delivery of radiation at various angles of a treatment beam as a linear accelerator rotates around the patient [45]. Using techniques of dynamic adjustments of multiple leaf collimator (MLC) positions, gantry rotation, and dose rate, it is possible to prescribe dose distributions highly conformal to complex target volumes, sparing the most sensitive critical structures such as the brain, brainstem, oral cavity, and parotid glands [46]. The lower monitor requirement and faster treatment delivery of VMAT increases patient comfortability, reduces intrafraction motion and improves efficiency, therefore is now emerged as one of the standard radiotherapy treatments for the HNCs.

#### Stereotactic radiosurgery (SRS) and Stereotactic radiotherapy (SRT)

Stereotactic radiosurgery is the highly precise radiotherapy technique that delivers a single high dose or few high dose fractions to small intracranial lesions using advanced image guidance. It is a non-invasive treatment, initially designed to treat small brain tumors and functional neurological disorders. The purpose is to deliver high radiation dose to eradicate the tumor while achieving durable local control [47]. Although SRS consists of a single treatment session, multiple stereotactic treatment sessions are more preferred for larger tumors (over approximately 1 inch in size). This is because to improve treatment safely and provide sufficient time for healthy tissues to recovery between treatment fractions. This technique is known as fractionated SRT, typically consists of two to five fractions of highly focused radiation delivered over non-consecutive treatment days [48]. In the care of patients with HNCs, its application is generally restricted to small, well-defined intracranial targets like skull-base tumours, cranial nerves, recurrent HNCs and brain metastases from primary HNC rather than for primary extracranial HNCs. Both SRS and SRT offer effective management of patients with local recurrence, achieving tumor control with a lower risk of cognitive toxicity.

#### Stereotactic body radiation therapy (SBRT)

An advanced non-invasive technique for delivering a high dose of targeted radiation therapy to a small, well-defined target and spare the surrounding tissue is SBRT. It delivers high doses of radiation to the tumor in a few fractions over a treatment course of one to two weeks. SBRT is primarily used for extracranial tumors, including selected HNCs rather than as a routine treatment for newly diagnosed disease [49]. Its principal applications include re-irradiation of recurrent HNCs, treatment of medically inoperable patients, small unresectable tumors, and management of small localized recurrences. This highly conformal radiation delivery is achieved through advanced imaging techniques including CT and MRI, which provide accurate tumor visualization and precise beam targeting [50]. SBRT can be particularly beneficial for patients who cannot tolerate lengthy treatment courses and reduces the likelihood of toxicities associated with conventional RT. Recent developments in treatment planning and radiation therapy technologies are improving the precision and efficiency of SBRT delivery for patients with HNC [51].

#### Tomotherapy

Another innovative technique in cancer care is Tomotherapy, a IGRT platform that combines helical IMRT with integrated daily megavoltage computed tomography (MVCT) imaging. The combination of these technologies allows the radiation beam to accurately target the tumor according to its shape, size and location [52]. During the treatment, exact location of the tumour and normal structures is verified with the help of a CT scan and these tumours, whether they are large or small, one or many, can be treated in the same session. In HNCs, Tomotherapy is particularly beneficial for patients with extensive primary tumours, or irregular target volumes located adjacent to multiple organs at risk (OAR) [53]. Compared to conventional radiotherapy, tomotherapy was found to be beneficial by improvement of OAR sparing and more homogeneous dose distributions.

#### Proton therapy (PT)

Proton therapy is another form of radiotherapy that utilizes proton particles in place of conventional photon beams. Its principal advantage lies on the characteristic Bragg peak, where protons deliver most of their energy at a specific depth, thereby minimising the exit dose outside the target region. It is increasingly used for selected patients with skull base tumours, HNCs, and cases requiring re-irradiation. In the treatment of HNCs, because of the close anatomical location of critical OARs, the radiation dose must be strictly limited [54]. Due to its unique physical properties, PT has the ability to reduce treatment-related toxicities and patient side effects while ensuring tumor control comparable to modern photon-based techniques [55]. So compared to conventional photon radiotherapy, PT improves the capability of protecting surrounding healthy tissue, but has the limitations in high costs of installation, availability and the need for specialized treatment facilities.

#### Adaptive radiotherapy (ART)

Although IMRT and VMAT are widely implemented for the treatment of HNCs in most healthcare settings, it has steeper dose fall-off beyond the target. These dose gradients signify that even small variations in the tumor volume, OAR location or anatomical changes, will have the effect of causing insufficient target coverage and increased dose to OARs [56]. During HNC treatment, anatomical modifications usually happen as from the initial therapy sessions, which includes the shrinking of tumors and even healthy tissues leading to organ movement and position changes relative to other structures. These minimal changes results in large variations in the dosimetric distribution. The one potential option to mitigate such events is called adaptive radiotherapy (ART), which necessitates re-scanning patient by CT and modifying plan according to the new anatomy [57].

#### Palliative radiotherapy

Although many advances in RT have improved the outcomes in curative-intent treatment, universal guidelines for palliative treatment remain lacking. Palliative care, “treatable but not curable cancers”, an integral component of supportive oncology, which aims to relieve tumour-related symptoms including pain, bleeding, dysphagia, airway obstruction, tumor ulceration or foul-smelling lesions, preserve organ function. It is commonly offered to minimise treatment burden in patient with locally recurrent, metastatic disease, and unresectable HNCs [58]. Compared with curative treatment, palliative radiotherapy generally involves shorter treatment courses and lower total radiation doses, allowing rapid symptom relief, reduction in tumor size, and treatment-related toxicity. In selected patients, modern treatment modalities like IMRT, VMAT, or SBRT may be used to optimize dose distribution and spare adjacent normal tissues, particularly in re-irradiation. Palliative radiotherapy is a cost-effective and widely available treatment modality, plays a pivotal role in effective symptom management and improved quality of life in patients with incurable HNC [59]. Although the advent of immunotherapy, particularly immune checkpoint inhibitors has improved survival rates and long-term protection with fewer side-effects, their high cost, limited affordability and availability for specialized healthcare settings continues to pose significant challenges in many low and middle-income countries, including India. Consequently, despite advances in immunotherapy, palliative radiotherapy is generally more affordable, accessible and effective option for symptom control and quality of life improvement in patients with advanced HNCs [60, 61]. Optimal outcomes of the treatment depend on appropriate patient selection, timely access to treatment, and comprehensive multidisciplinary supportive care.

Advancements in radiation therapy are vital to overcome challenges in treating HNCs to improve therapeutic outcomes and reduce adverse effects [62]. Tumor complexity and anatomical challenges arise because HNCs often occur near vital structures like the spinal cord, salivary glands, and optic nerves; advancements in radiotherapy enable precise tumor targeting, reducing radiation dose to these critical organs and lowering the risk of severe side effects. Personalization of treatment through integration of radiomics and artificial intelligence enables individualized planning by analysing imaging data to predict tumor response and toxicity risk, moving beyond a one-size-fits-all approach and improving both efficacy and safety [63]. Technological advances in imaging, treatment delivery, and computing power make it possible to monitor in real-time and adjust treatments during radiotherapy, which increases treatment precision and patient safety. Overall, these improvements address the limitations of traditional radiation therapy, boost tumor control, minimize treatment-related toxicity, and ultimately enhance survival and quality of life for patients with HNCs [62].

## Conclusion

Cancer is still a major global public health challenge, with its burden predicted to rise substantially due to population growth and aging. Current estimates suggest that by 2050, global incidence will reach 35 million, with Asia accounting for nearly half of this burden. In this scenario, HNC emerges as a particularly serious type of cancer. A comparative analysis shows that India’s HNC profile is notably different from the region’s; for instance, the ASIR for lip and OCC in India is 9.8, more than three times the Asian average of 3.2 and exceeding the global average of 4.1.

This review highlights that India has one of the highest HNC burdens in the world, characterized by a stark gender disparity. Indian men are facing a critical public health crisis with an ASIR of 14.7 for OCC. This rate is nearly double the 4.9 ASIR observed in women. The ASMR is also high at 8.1 for men and 2.9 for women across the country. These figures, along with a much lower burden of nasopharyngeal cancer in India (ASIR 0.4) compared to the rest of Asia (ASIR 1.9), highlight the region’s specific health challenge. Such high MIR across all sub-sites indicate many cases are diagnosed at an advanced stage. This situation emphasizes the urgent need for focused prevention and early detection programs.

Radiotherapy has changed significantly and is still a remains a cornerstone of managing HNC. The transition from conventional 2DRT to 3DCRT, IMRT, IGRT, VMAT and advanced modalities like SRS, SRT, SBRT, tomotherapy, proton therapy and adaptive therapy has substantially improved dose conformity and sparing of organs at risk. Despite these advances, precise and reproducible patient positioning is crucial to the clinical efficacy of radiation therapy, especially in addressing the high mortality rates noted above.

In conclusion, a comprehensive strategy that integrates prevention, early diagnosis, and technologically advanced treatment delivery is needed to address the rising burden of HNC. Continued research focused on optimizing radiotherapy techniques and improving positioning accuracy with 6DOF systems will be essential to improve survival outcomes and quality of life for HNC patients in the years ahead.

## Data Availability

Data sharing is not applicable to this article as no new data were created or analysed in this study. This review is based entirely on previously published literature, all of which is cited in this article.
